# International Hip Outcome Tool (12-items) as health-related quality-of-life measure in osteoarthritis: validation of Greek version

**DOI:** 10.1186/s41687-020-00207-8

**Published:** 2020-05-27

**Authors:** Sophia Stasi, Magdalini Stamou, George Papathanasiou, Paraskevi Frantzeskaki, Emmanouil Kanavas, George Evaggelou-Sossidis, Adamantios Gouskos, Andreas Palantzas, Kyriakos Poursanidis, George A. Macheras

**Affiliations:** 1 Laboratory of Neuromuscular and Cardiovascular Study of Motion, Physiotherapy Department, Faculty of Health and Care Sciences, University of West Attica, Campus 1, 28 Agiou Spyridonos St., 12243 Egaleo, Attica Greece; 2 4th Department of Orthopaedics, “KAT” General Hospital of Attica, 2 Nikis St., Kifissia, 14561 Athens, Greece

**Keywords:** Factor analysis, Reliability, Validity, Cut-off points, Responsiveness, Direct anterior approach - minimal invasive surgery

## Abstract

**Background:**

The 12-item International Hip Outcome Tool (iHOT12) is a patient-reported outcome (PRO) designed to evaluate quality of life. We assessed the psychometric properties of the Greek version (iHOT12-Gr) in hip osteoarthritic patients.

**Methods:**

Data from 124 patients aged > 50 years were used for factor analysis. Reliability evaluation included internal consistency, test-retest reliability, and interpretability. Content validity was examined by calculating the item-level content validity indices (I-CVI) and the scale-level content validity indices (S-CVI), using two methods: S-CVI Average (S-CVI/Ave), and the S-CVI Universal Agreement among experts (S-CVI/UA). Construct validity was tested against Greek versions of the Lower Extremity Functional Scale (LEFS-Greek), Modified Harris Hip Score (MHHS-Gr), and the 30 s chair-to-stand, Timed Up & Go (TUG), and 9-stairs-ascend/descend (9S-A/D) tests. Known-groups validity was examined using LEFS-Greek (cut-off = 53 points) as estimate variable. Responsiveness was examined pre and post total hip arthroplasty (4 and 8 weeks).

**Results:**

*Factor analysis* revealed a two-factor model. Factor-1 (items 1–9) reflects “Symptoms and functionality”, while Factor-2 (items 10–12) reflects “Hip disorder-related concerns”. *Reliability*: Internal consistency and test-retest reliability of iHOT12-Gr-total were excellent: Cronbach’s alpha > 0.92 and ICC(95% CI) > 0.976(0.96–0.99)(*p* < 0.001). *Interpretability*: There was no floor or ceiling effect; measurement error: 3.72 (Factor-1), 3.64 (Factor-2), and 3.22 (iHOT12-Gr-total); minimal detectable change: 10.3 (Factor-1), 10.1 (Factor-2), and 8.92 (iHOT12-Gr-total). *Validity:* Content validity: The I-CVI value of the 12 items ranged from 1.00 to 0.83, the S-CVI/Ave was 0.97 and the S-CVI/UA was 0.83. Construct validity: iHOT12-Gr correlated strongly with both LEFS-Greek and MHHS-Gr, and weakly but significantly with 30s chair-to-stand, TUG and 9S-A/D (*p* < 0.001). Known-groups validity showed that iHOT12-Gr well discriminated subgroups of patients (*p* < 0.001). ROC analysis cut-off points were 51.9 (Factor-1), 25 (Factor-2) and 45.2 (iHOT12-Gr-total) (*p* < 0.001). *Responsiveness*: Four and 8 weeks postoperatively, standardized response means of Factor-1, Factor-2, and iHOT12-Gr-total were > 0.8.

**Conclusion:**

iHOT12-Gr showed excellent reliability properties. The content validity was excellent and significant weak-to-strong correlations were found regarding construct validity. The known-group validity was also significant, while the responsiveness was excellent. iHOT12-Gr could be a reliable and valid PRO for assessing quality of life in patients with hip osteoarthritis.

## Introduction

Hip osteoarthritis (OA) is among the most prevalent and disabling conditions affecting the elderly [1]. Worldwide, there is an estimated 25% lifetime risk of symptomatic hip OA in people who live to age 85 [2], and an almost 10% lifetime risk of undergoing total hip arthroplasty (THA) for end-stage OA [3]. In the Greek population, hip OA has a prevalence of 0.9/1000: 1.5/1000 in women and 0.3/1000 in men [4]. As the prevalence and incidence of the disease continues to rise, the proper measurement of OA severity and its impact on health status becomes a crucial component in any orthopaedic or clinical practice [5]. Aligning with a patient-centred healthcare delivery model, the quality and success of interventions aiming to treat OA should be based on outcomes deemed imperative by the patients [5]. Hence, measurement instruments applied in the clinical setting should include patient-reported outcome (PRO) measures. PROs are considered ideal measurement tools for evaluating outcomes [6] because patients can participate actively in their own evaluation [7].

The symptomatic manifestations of OA as a combination of pain and stiffness contribute substantially to functional disability, significantly decreasing the patient’s quality of life (QoL) [8]. Health-related QoL can be evaluated using a generic PRO to assess the impact of the disease on a patient’s general health, or a specific PRO to measure only the impact of the disease on the QoL domains affected directly by the disease itself [9].

The 12-item International Hip Outcome Tool (iHOT12) is a specific QoL PRO that was developed in 2012 by Griffin et al. [10] as a shorter version of the 33-item International Hip Outcome Tool questionnaire [11]. Both questionnaires were designed to measure the impact of hip disorders on the QoL of young, active patients. The original English version of iHOT12 was cross-culturally adapted for Portuguese patients [12], while Swedish [13], Dutch [14], German [15], Japanese [16] and Turkish [17] versions of the questionnaire were also proved reliable and valid. Despite the widespread use of iHOT12 in clinical research and practice worldwide, there is a lack of information regarding its applicability in older patients with chronic hip diseases, such as hip OA. The need for a joint-specific PRO to evaluate the QoL in older patients, one that is short and easily implemented in clinical practice, led us to adapt the iHOT12 questionnaire for Greek patients.

The purpose of the present study was to examine the reliability, validity, and responsiveness after THA, of iHOT12 in Greek individuals with hip OA. Additionally, we set out to conduct factor analysis and to define the cut-off points of the PRO in hip OA patients. Examination of the psychometric properties of the cross-culturally adapted Greek version of the iHOT12 questionnaire would allow its broader clinical use in hip OA patients and could add to the overall value of the instrument. A broader awareness of these findings in the Greek setting would facilitate objective comparisons between studies with different national origins and could contribute to the validity of future meta-analyses.

## Material and methods

This observational study was conducted in accordance with the 1964 Helsinki declaration and its later amendments [18]. The Scientific Research Council of the “KAT” General Hospital of Attica, Athens, Greece approved the protocol (ref: No5/13-02-2018). The study conformed to the “*Strengthening the Reporting of Observational studies in Epidemiology*” (STROBE) statement for reporting observational studies [19].

### Description of iHOT12

The iHOT12 instrument is a joint-specific PRO for evaluating QoL. The questionnaire includes 12 questions from the original iHOT33, related to the patient’s symptoms, limitations and concerns. The 12 items of iHOT12 are included in the Additional file 1. Like iHOT33, iHOT12 is divided into four factors (sections). Factor-1 “*symptoms and functional limitations*” (items: 1, 2, 3, 4), Factor-2 “*sports and recreational activities*” (items: 6, 7, 11), Factor-3 “*job-related concerns*” (item: 5), and Factor-4 “*social, emotional, and lifestyle*” (items: 8, 9, 10, 12). The patient is asked to consider the problems arising from his/her hip disorders and to quantify the level of his/her QoL on a 100 mm horizontal line (visual analogue scale) by marking it with a slash. Each question has equal weight, giving a mean score from 0 to 100. The 12 items are scored together, rather than the 4 factors separately. A score of 100 indicates excellent QoL (full function and no symptoms), whereas zero signifies the worst QoL (maximum limitations and extreme symptoms) [10].

### Cultural adaptation phase

Official permission for reprinting and translating the English/original iHOT12-Gr questionnaire was granted by Professor Damian R. Griffin. Its adaptation into Greek followed the guidelines developed by Guillemin et al. [20, 21] and Beaton et al. [22]. Technical and linguistic adaptations were carried out by a team of experts (health science professionals and two bilingual non-medical specialists). During this phase, item 9 (“*How much trouble do you have with sexual activity because of your hip*?”) was slightly controversial. In the original questionnaire this item had the option “*This is not relevant to me*”, essentially allowing participants not to answer. As mentioned above, iHOT12 included 12 items from the original iHOT33, divided into four factors (sections) [10]. Since it was reported that in short version questionnaires 3 or 4 items should be included for each section [23], it was suggested that all questions should be answered when iHOT12 was implemented [24]. Moreover, in the iHOT12 instructions page patients are required to answer all questions, imagining how their hip would feel even if they had not performed that activity [10]. For these reasons, we decided to remove the option “*This is not relevant to me*” from the Greek version of iHOT12. It is worth noting that none of the group of individuals (*n* = 20) who completed the provisional version, or the two groups (*n* = 15) who completed the final version, commented on this item or showed discontent while answering. These three groups consisted of individuals with the same demographic and clinical characteristics as the participants in our study. They were hip OA patients who had been consulting the senior orthopaedic surgeon who was among the researchers of the present study. The questionnaire was administered to them by means of one-to-one interviews during their visit to the hospital.

The back-translation was approved by the creator of the original, Professor Damian R. Griffin. The original questionnaire, the Greek-language version and the back-translation of iHOT12-Gr are included in the Additional file 1.

### Validation phase

#### Participants

Between February and December 2018, 165 patients aged 50 years and over, who came to the hospital to consult the senior orthopaedic surgeon who is an author of the present study, were evaluated for participation. The main inclusion criterion was the existence of hip OA according to the Kellgren–Lawrence classification system [25]. Patients who reported pain on active movement of the hip joint and had used anti-inflammatory medication and/or received physiotherapy for at least the previous 6 months were eligible for inclusion [26]. Participants were excluded if they had other types of arthritis, or lower-limb muscle weakness due to a central or peripheral neurological aetiology, or declared insufficient knowledge of the Greek language. If participants reported any change in their clinical status or received any treatment between the two assessment days, they were also excluded from the reliability analysis. Upon acceptance, participants gave their written informed consent and their demographic and clinical characteristics were recorded.

#### Procedures

In the present study, content validity was determined using a panel of experts. The recommended number of experts varies from 3 to 10; a panel of 6 experts is considered adequate [27]. The iHOT12-Gr questionnaire was evaluated by 6 experts: three senior orthopaedic surgeons and three physiotherapists, who had two decades of expertise in the lower limbs as well as ample research experience. To avoid subjectivity bias, they were not involved in any part of the study. The experts were asked to rate each iHOT12-Gr item in terms of its relevance for evaluating the impact of hip OA on a patient’s QoL domain [27], using a 4-point Likert scale: 1 = not relevant, 2 = somewhat relevant, 3 = quite relevant and 4 = very relevant [28]. Ratings of 1 and 2 were considered as content invalid, while ratings of 3 and 4 were considered content valid [27].

To explore the construct validity properties of iHOT12-Gr, the Greek versions of the PROs Lower Extremity Functional Scale (LEFS-Greek) [29–31] and Modified Harris Hip Score (MHHS-Gr) [32, 33], and the physical-performance measures (PPMs) 30s chair-to-stand [34], Timed Up & Go (TUG) [35] and 9-stairs-ascend/descend (9S-A/D) [36] were used for comparison. These PROs and PPMs are described in detail below.

Regarding patients’ measurements, on the initial assessment (day-1), iHOT12-Gr, LEFS-Greek and MHHS-Gr were given to all participants and completed on site, under the supervision of the same member of the research team. The questionnaires were given out in random order, interspersed with the PPMs (one questionnaire – 30 s chair-to-stand test – one questionnaire – TUG test – one questionnaire – 9S-A/D test). This allowed sufficient resting time between the tests and reduced the risk of question-order bias. The correct procedures for the 30 s chair-to-stand, TUG and 9S-A/D were carefully explained prior to a single pilot test. PPMs were performed only once, so as to minimise habituation bias and avoid affecting the participant’s performance. The PPM performance times of a patient were recorded only by one of the three senior physiotherapists of the present study, using the same timer, with an accuracy of 1/100 s. Participants were allowed to use a walking aid if necessary, but no verbal encouragement or personal assistance was given. Participants were asked to perform the 30-s chair-to-stand test and the TUG test as quickly as they could while still feeling safe, while for the 9S-A/D test they were asked to proceed in their usual manner, at a safe and comfortable pace, using the stair’s handrail if necessary. In the present study, the times taken to ascend and descend the stairs were measured separately and the total time in seconds was recorded. Seven days after the first assessment day (day-8), during a scheduled appointment, one researcher visited the patients at their homes and the iHOT12-Gr questionnaire was re-administered.

Patients from our study population who were on the waiting list for THA via direct anterior approach minimally invasive surgery (DAA-MIS) were used to explore the questionnaire’s responsiveness (treatment effect validity). This sub-sample of participants completed the iHOT12-Gr questionnaire on a further three occasions: preoperatively (the day before surgery), 4 weeks after DAA-MIS during the first routine postoperative appointment with their surgeon, and during the 8th week after DAA-MIS during another scheduled in-home appointment.

#### Patient-reported outcomes and physical performance measures

##### Lower extremity functional scale

The Lower Extremity Functional Scale is a functional status PRO that aims to investigate the degree of difficulty an individual experiences in performing everyday tasks [29]. The questionnaire has 20 items, each of which is scored from 0 (Extreme difficulty or unable to perform activity) to 4 (No difficulty). Answers are summed and reported as a total (0–80), with higher scores reflecting greater self-perceived functional ability. In the present study, the reliable [30], and valid [31] Greek version of the Lower Extremity Functional Scale questionnaire (LEFS-Greek) was used.

##### Modified Harris hip score

The Modified Harris Hip Score (MHHS) is a patient-reported questionnaire that includes assessments based on pain and on function. One item evaluates the pain (0–44 points), while 7 items evaluate the patient’s functionality (0–47 points). The total points form a scale from 0 to 91. A multiplier of 1.1 provides a total score of 100 (best possible outcome) [32, 33].

##### 30 s chair-to-stand test

The 30 s chair-to-stand test provides a measurement of a person’s lower limb strength. The procedure involves recording the number of stands a person can complete in 30 s from a folding chair, with a seat-height of 17 in. (43.2 cm) without armrests. It is associated with the ability to perform lifestyle tasks such as climbing stairs, or getting in and out of the bath [34].

##### Timed up & go (TUG) test

The Timed Up & Go (TUG) test was introduced in 1991 by Podsiadlo and Richardson. It is a simple, rapid and widespread clinical tool for the measurement of functionality and mobility. The TUG test measures the time (in seconds) taken by a participant to stand up from an armed chair with a seat height of 46 cm, walk for 3 m, turn around a cone and return to sit on the same chair. A shorter performance time represents better functionality [35].

##### 9-stairs-ascend/ descend test

The 9-stairs-ascend/ descend (9S-A/D) test was developed for end-stage hip and knee OA patients. It is an excellent functional measure because stairs are relevant to people’s daily-life activities and have been related to independence and community participation. This test measures the time (s) needed to ascend and descend a flight of 9 stairs with a step height of 20 cm. A shorter performance time represents better functionality [36].

### Statistical analyses

For the exploration of psychometric properties of PRO questionnaires, there is a widely-cited rule of thumb that suggests 10 respondents per item [37, 38]. The iHOT12 questionnaire consists of 12 items; thus a sample size of 120 participants would be adequate, while a sub-sample size of 50 participants is considered adequate for determining test-retest reliability [39].

All tests were two-sided, a *p*-value < 0.05 was considered to denote statistical significance. All analyses were carried out using the statistical package SPSS version 17.00 (Statistical Package for the Social Sciences, SPSS Inc., Chicago, Ill., USA). Data were expressed as Mean ± SD for quantitative variables and as percentages for qualitative variables.

### Factor analysis

Confirmatory factor analysis (CFA) was used first, to examine the factor-structure of the questionnaire suggested by its creator [10]. The CFA was carried out using the Analysis of Moment Structure (AMOS) Version 21.0. Rejecting or accepting a model was based on the global fit indices: chi-square degrees of freedom (d.f.) ratio, root mean square error of approximation (RMSEA), comparative fit index (CFI), normed fit index (NFI), goodness fit index (GFI), and adjusted GFI (AGFI). A chi-square d.f. ratio < 2.0, RMSEA< 0.08, CFI > 0.90, NFI > 0.90, GFI > 0.85 and AGFI> 0.80 indicate an acceptable fit [40].

Exploratory factor analysis (EFA), using a maximum likelihood extraction method with oblique rotation, was conducted for all participants to determine the latent factor structure of the iHOT12-Gr questionnaire. Factor adequacy was assessed using Bartlett’s sphericity and the Kaiser–Meyer–Oklin (KMO) tests. Items with factor loadings ≥0.40 (including values that rounded to 0.40) and those that did not load on more than one factor were retained. Items not meeting these criteria were removed one at a time. Factor analyses were repeated until a solution was attained in which all items included in the analysis met all criteria. The number of factors retained from the EFA was also confirmed using a Monte Carlo principal component analysis (PCA) [41].

Item analysis of the iHOT12 questionnaire was performed by analysing the item discriminating power (corrected item correlation) and the item difficulty (item mean) depicted by the explanatory data analysis.

### Reliability study

A reliability study was carried out to explore the internal consistency and test-retest reliability (stability) of iHOT12-Gr. The internal consistency was determined by calculating Cronbach’s *alpha* coefficient [42]. The test-retest reliability was estimated by calculating the intraclass correlation coefficient (ICC) and its 95% confidence interval (CI) [43]. Because this coefficient does not correct for systematic differences and agreement by chance, the scores of the two assessments (day-1 and day-8) were tested for systematic differences using the paired *t*-test. Finally, a Bland–Altman plot was used as a visual method of assessing stability [44].

Interpretability/repeatability refers to the degree to which one can assign qualitative meaning to quantitative scores [6]. It was determined by calculating the floor and ceiling effects, which are considered to be present if more than 15% of respondents achieve the lowest or highest possible score [43].

The measurement error is the error of the score not attributable to the construct being measured and is expressed as the standard error of measurement (SEM), using the formula SEM = SD × √(1–ICC), where SD is the standard deviation of all patients at baseline. Minimal detectable change (MDC) is the change of score that exceeds the SEM and was calculated as SEM × 1.96 × √2 at the individual level [45].

### Validity study

Content validity measures how well items correspond to or reflect a specific domain [46]. The most widely reported approach for content validity is the index of content validity (CVI), which refers to the degree to which an instrument has an appropriate sample of items for the construct being measured, and is obtained by calculating the item-level content validity indices (I-CVI) and the scale-level content validity indices (S-CVI) [46]. I-CVI measures the proportion of content experts giving the item a relevance rating of 3 or 4 (content validity of individual items), while the S-CVI is the content validity of the overall scale [46]. There are two methods for calculating S-CVI: the Average CVI (S-CVI/Ave) and the Universal Agreement (UA) among experts (S-CVI/UA). S-CVI/Ave is calculated by taking the sum of the I-CVIs divided by the total number of items, while S-CVI/UA is calculated by adding all items with I-CVI equal to 1.00 divided by the total number of items [27]. Regarding the I-CVI values: > 0.79 means the item is relevant, 0.70–0.79 the item needs revision and < 0.70 the item had to be deleted. S-CVI/UA values ≥0.8 and a S-CVI/Ave values ≥0.9 indicate excellent content validity [27].

Construct validity was defined as the degree to which an outcome score is consistent with another relevant score [6]. Spearman’s correlation coefficient was used to interpret the data [47]. A Spearman correlation value 1.0–0.80 is characterised as “*very strong*”, 0.79–0.60 as “*strong*”, 0.59–0.40 as “*moderate*”, 0.39–0.20 as “*weak*”, and 0.19 to 0.00 as “*very weak*” [48]. A strong and significant correlation (0.60–0.79) between iHOT12-Gr and the well-established PROs and objective PPMs would validate the iHOT12-Gr questionnaire for measuring important aspects of functional status in hip OA patients.

The known-groups validity was examined in terms of iHOT12-Gr’s ability to distinguish between subgroups of patients formed on the basis of their functional status according to the cut-off point (53 points) of LEFS-Greek [31]. An independent samples *t*-test was used for the statistical analysis.

Receiver operating curve (ROC) analysis was conducted to determine the cut-off point of iHOT12-Gr for differentiation between subgroups of patients formed on the basis of their functionality. The area under the curve (AUC), standard error and 95% CI were calculated using the maximum likelihood estimation method, and the sensitivity and specificity of different cut-off points for iHOT12-Gr as a measure of QoL were estimated using the cut-off point (53 points) of LEFS-Greek as estimated variable [31].

### Responsiveness study

Responsiveness (treatment effect validity) was examined in terms of the questionnaire’s ability to monitor changes after THA surgery. It was determined using the one-way repeated measures model between the iHOT12-Gr total scores at baseline and at the 4th and 8th postoperative weeks, calculating the standardized response mean (SRM) using the formula SRM = Mean_Postoperative_–Mean_Preoperative_ / Standard deviation_Postoperative–Preoperative_ [49]. For characterizing the SRM findings, we used the threshold level of Cohen’s effect size, which suggests that an absolute value of 0.8 or greater indicates excellent responsiveness [50].

## Results

### Descriptive and clinical data

**Fig. 1:**
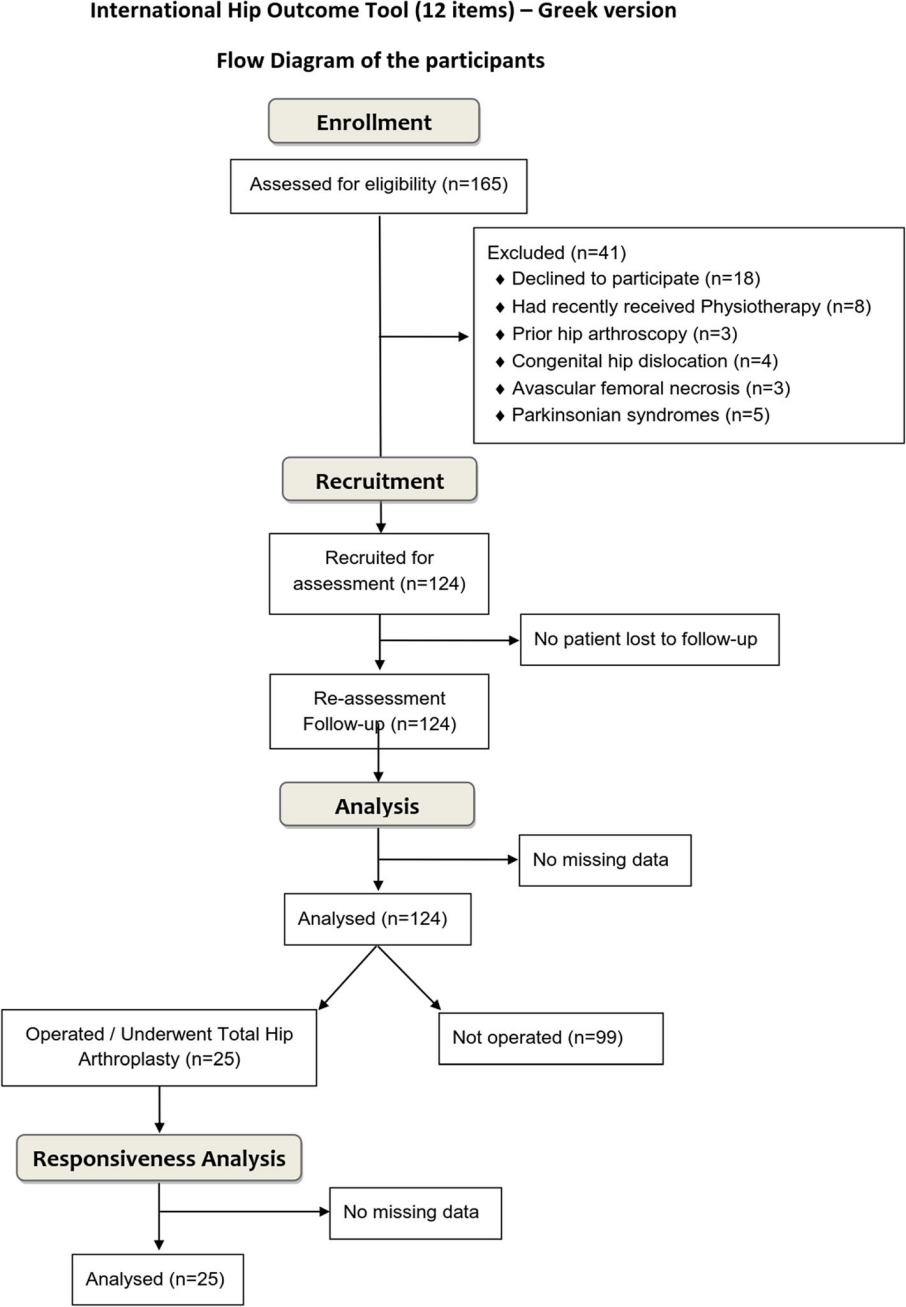
Flow diagram of the participants’ enrolment

The data from 124 participants were analysed (Fig. 1). Regarding the responsiveness of iHOT12-Gr after treatment, data from a sub-sample (*n* = 25) of our participants who were suffering from a late stage of hip OA and underwent THA through DAA-MIS were analysed. This phase of the study lasted from May until December 2018 (Fig. 1). The participants’ demographic characteristics and clinical measurements are shown in Table 1.

**Table 1:** Demographic and clinical characteristics of the study’s participants (*n* = 124)

Characteristics	Values^a^
Age (years)	65.80 ± 8.25 (50–85)
Sex, n (%): men/women	29 (23%) / 95 (77%)
Height (m)	1.64 ± 0.08 (1.50–1.83)
Weight (kg)	76.14 ± 15.57 (48–135)
Body mass index (kg/m^2^)	28.07 ± 4.77 (17.4–45.6)
Dominant, n (%): right/left	104 (84%) / 20 (16%)
Affected hip, n (%): right/left	68 (55%) / 56 (45%)
Nocturnal pain, n (%): no/yes	61 (49%) / 63 (51%)
Morning stiffness, n (%): no/yes	48 (39%) / 76 (61%)
Assistive device, n (%): no/yes	100 (81%) / 24 (19%)
Kellgren–Lawrence classification of hip osteoarthritis, n (%): Grade 1/2/3/4	5 (4.0%) / 18 (14%) / 64 (52%) / 37 (30%)
International hip outcome tool (12 items): Greek version’s total score (initial assessment)	38.17 ± 23.99 (3.3–95.8)
Lower extremity functional scale: Greek version’s total score	40.64 ± 17.84 (4.0–80.0)
Modified Harris hip score: Greek version’s total score	61.4 ± 18.76 (5.3–51.8)
30 s chair-to-stand test (rep)	8.00 ± 2.66 (2.0–18.0)
Timed Up and Go test performance time (s)	14.55 ± 7.86 (6.1–45.3)
9stairs-Ascent/Descent test performance time (s)	19.71 ± 9.56 (5.3–51.8)

^a^The values are expressed as Mean ± Standard deviation (SD) and Range for continuous variables and as frequencies (n) and percentages (%) for categorical variables

### Factor analysis

**Fig. 2:**
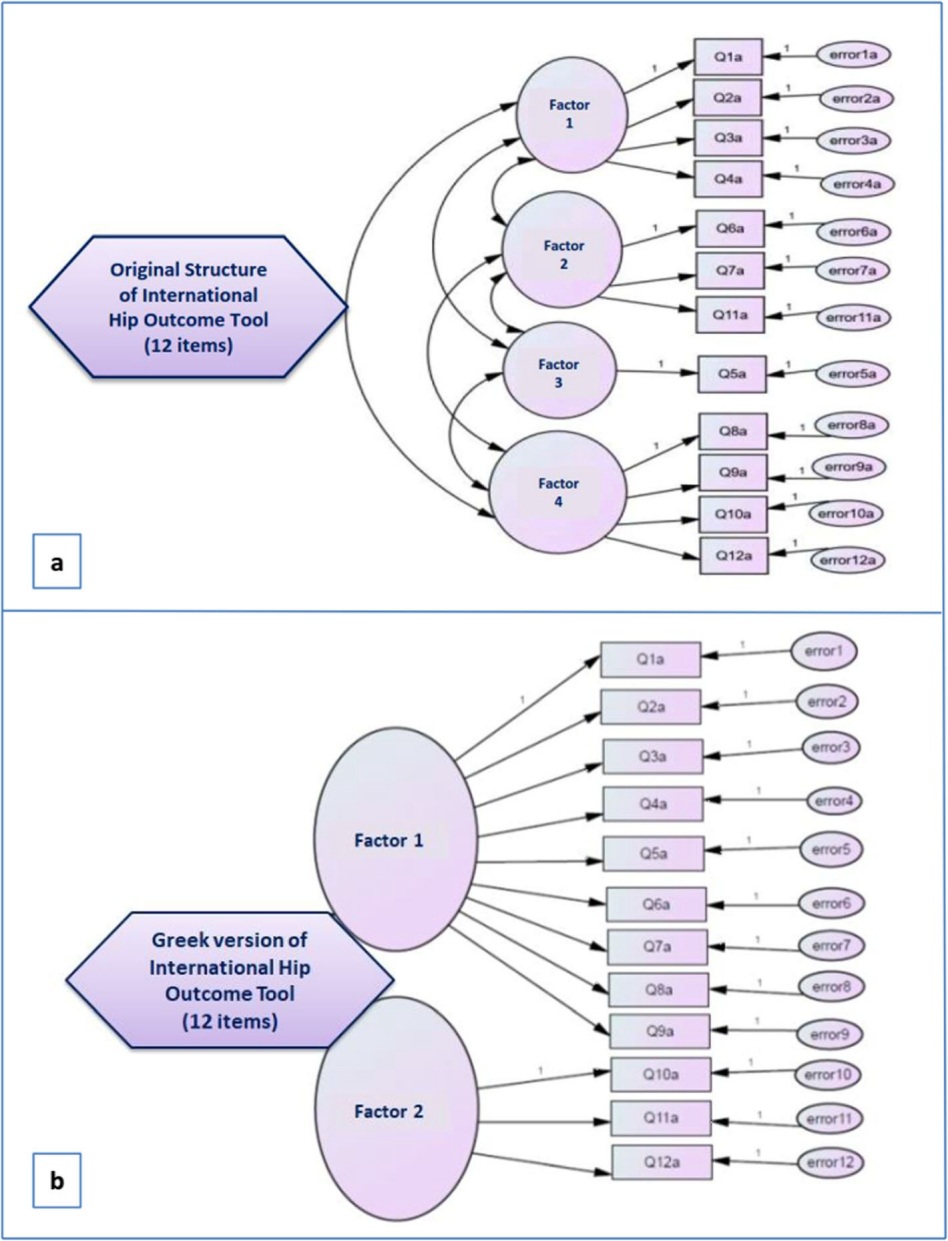
**a** The four-factor model of the International Hip Outcome Tool–Greek version based on the original structure gave unacceptable global fit indices; **b** The two-factor model gave acceptable global fit indices: X^2^ = 103.3, chi-square-degrees of freedom (d. f.) ratio = 1.95, RMSEA = 0.073, CFI = 0.927, NFI = 0.893, GFI = 0.865, AGFI = 0.802

A four-factor model of iHOT12-Gr was first constructed using CFA (Fig. 2a), but gave unacceptable global fit indices. The resulting global fit indices, X^2^ = 188.2, chi-square d.f. ratio = 3.7, RMSEA = 0.148, CFI = 0.835, NFI = 0.790, GFI = 0.791 and AGFI = 0.680, showed that the four-factor solution proposed by the author should be rejected.

In the EFA, the Bartlett Test of Sphericity was 860.3 and was significant (*p* < 0.001).The Kaiser–Meyer–Olkin Measure of Sampling Adequacy was 0.901, showing suitable data for factor analysis [41]. The 12 items were analysed via maximum likelihood extraction method using an oblique rotation. Two factors were identified with eigenvalues > 1 and item factor loadings ≥0.40. The scree test and Monte Carlo PCA for parallel analysis (the criterion value was 1.35, higher than the eigenvalue of the third factor) confirmed the two-factor solution. The eigenvalues, explained variance and factor loadings are presented in Table 2.

**Table 2:** Factor analysis of the international Hip Outcome Tool (12 items) - Greek version questionnaire (*n* = 124)

Eigenvalues and explained variance				Factor loadings^a^	
Items	Eigenvalues	% of Variance	Cumulative %	Factor 1	Factor 2
1	***6.166***	51.380	51.380	.452	
2	***1.379***	11.492	62.873	.727	
3	.896	7.470	70.342	.752	
4	.747	6.228	76.570	.547	
5	.584	4.866	81.436	.769	
6	.573	4.774	86.210	.498	
7	.398	3.318	89.528	.608	
8	.334	2.785	92.314	.842	
9	.260	2.170	94.483	.683	
10	.251	2.090	96.574		.940
11	.231	1.929	98.503		.781
12	.180	1.497	100.000		.568

^a^Extraction method: maximum likelihood. Rotation: oblique with Kaiser normalization (rotation converged over 7 iterations). Only loadings with values > 0.40 are presented

A two-factor model of the iHOT12-Gr questionnaire based on EFA was examined by CFA, giving acceptable global fit indices. The resulting global fit indices, which are presented in Fig. 2b, showed that the two-factor solution proposed by the EFA should be retained.

Since factor analysis revealed a two-factor solution, the psychometric properties of the Greek version questionnaire were explored and presented for Factor-1, Factor-2 and the questionnaire’s total (iHOT12-Gr-total).

**Fig. 3:**
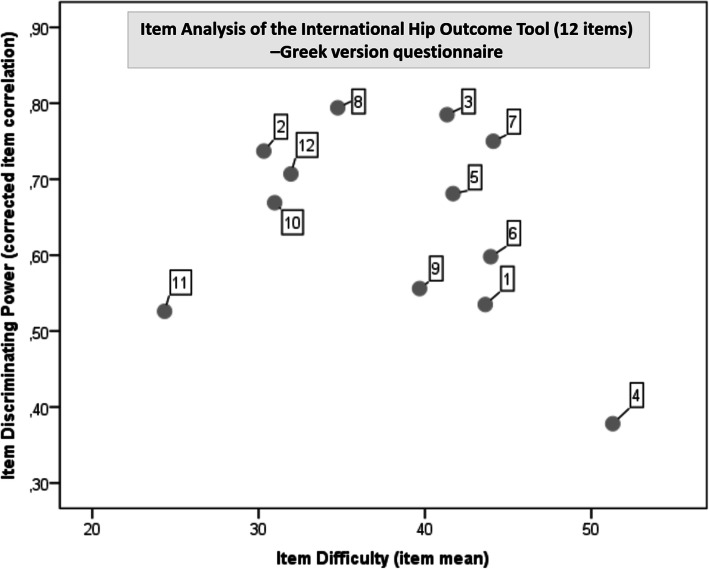
The item discriminating power (corrected item correlation) and the item difficulty (item mean) depicted by the explanatory data analysis of International Hip Outcome Tool–Greek version

The item analysis of iHOT12-Gr showed that item 8 had the highest corrected item correlation (0.794), whereas item 4 had the lowest (0.378). Item 11 had the lowest item mean (24.35) and item 4 the highest (51.29) (Fig. 3).

### Reliability properties

The internal consistency of Factor-1, Factor-2 and iHOT12-Gr-total was measured with Cronbach’s alpha, which indicated high and excellent internal consistency (Table 3).

**Table 3:** Reliability properties of the international Hip Outcome Tool (12 items) - Greek version

Internal consistency (*n* = 124)		Cronbach’s *alpha*		
	Factor-1	0.892		
	Factor-2	0.865		
	iHOT12-Gr-total^a^	0.907		
Test-retest reliability (*n* = 50)		ICC^b^95%CI^c^		*p*-value
	Factor-1	0.976 (0.96–0.99)		< 0.001
	Factor-2	0.987 (0.98–0.99)		< 0.001
	iHOT12-Gr-total^a^	0.982 (0.97–0.99)		< 0.001
		Paired samples *t* -test		*p*-value
		Initial assessment	Re-assessment	
	Factor-1	42.51 ± 23.52^d^	43.40 ± 23.50^d^	0.232
	Factor-2	33.50 ± 34.62^d^	33.63 ± 34.51^d^	0.865
	iHOT12-Gr-total^a^	40.26 ± 23.94^d^	40.96 ± 24.12^d^	0.282
Interpretability/Repeatability (*n* = 124)		Standard error of measurement	Minimal Detectable Change	
	Factor-1	3.72	10.3	
	Factor-2	3.64	10.1	
	iHOT12-Gr-total^a^	3.22	8.92	

^a^international Hip Outcome Tool (12 items) – Greek version Total

^b^Intraclass correlation coefficient

^c^Confidence interval

^d^The values are expressed as Mean ± Standard deviation (SD)

**Fig. 4:**
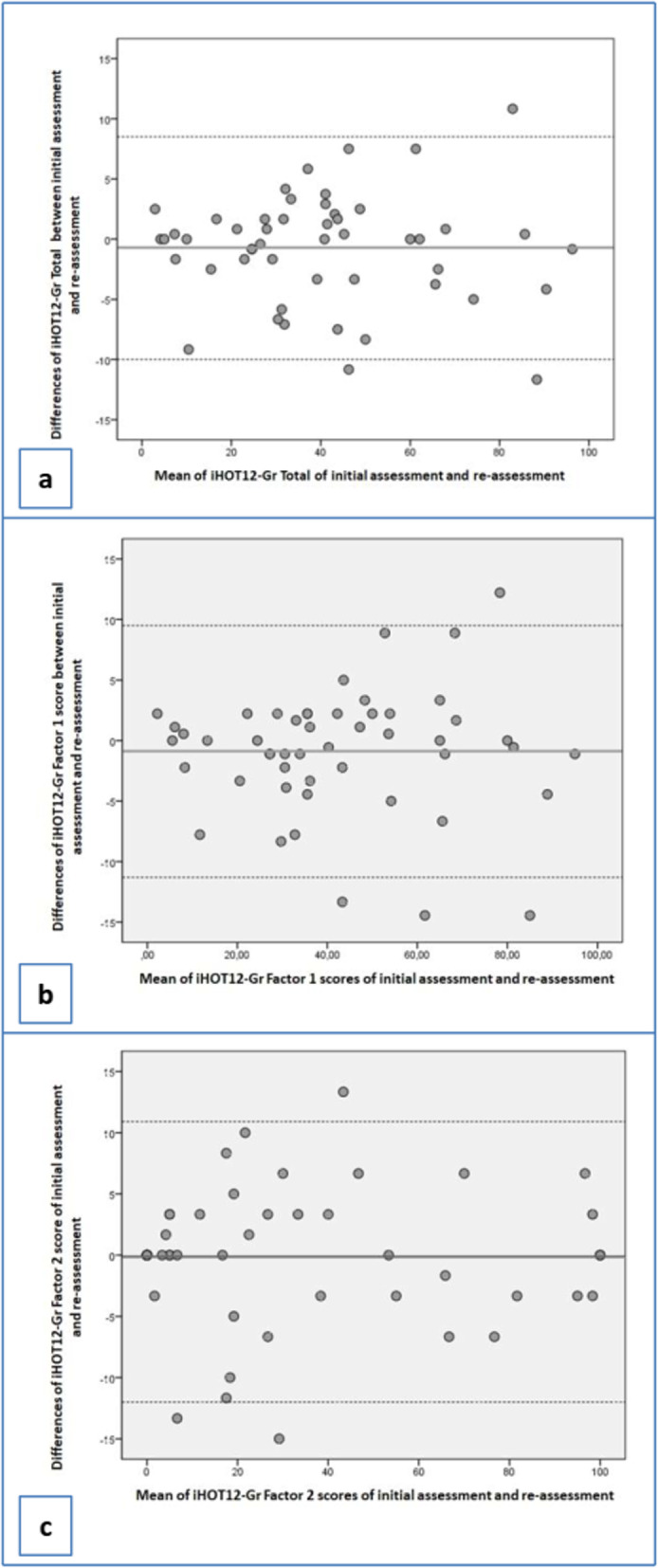
Bland–Altman plots showing difference in means between initial assessment and reassessment of: **a** International Hip Outcome Tool–Greek version Total, **b** Factor-1, and **c** Factor-2

As regards test-retest reliability, the ICC (95% CI) between the initial assessment and re-assessment of Factor-1, Factor-2 and iHOT12-Gr-total was in all cases > 0.976(0.96–0.99) (*p* < 0.001) (Table 3). The paired samples *t*-tests between initial assessment and re-assessment indicated no statistically significant difference (Table 3). Bland–Altman plots are presented in Fig. 4; inspection of the scattergram showed that almost all differences were within ±2 SDs [44], thus confirming the agreement between the two assessments.

The floor and ceiling effects were 0.8% and 1.6% for Factor-1, 1.5% and 5.6% for Factor-2, and 0.8% and 0.8% for iHOT12-Gr-total. The critical value of 15% was not surpassed [43], indicating no floor or ceiling effect for iHOT12-Gr. SEM and MDC values for Factor-1, Factor-2 and iHOT12-Gr-total are presented in Table 3.

### Validity properties

The results concerning the content validity of iHOT12-Gr showed that I-CVI values were above 0.79, while the values of S-CVI/Ave and S-CVI/UA were above ≥0.9 and ≥ 0.8, respectively. Overall, all items were found to be relevant, indicating that iHOT12-Gr has excellent content validity to measure QoL in elderly hip OA patients (Table 4).

**Table 4:** Content validity: ratings of iHOT12-Gr items by six experts on a 4-point scale^a^

Item	Expert 1	Expert 2	Expert 3	Expert 4	Expert 5	Expert 6	Number of Experts in agreement (items rated 3 or 4)	Item-level Content Validity Index
1	4	4	4	4	4	4	6	1.00
2	4	3	4	4	3	4	6	1.00
3	4	4	4	4	4	4	6	1.00
4	4	4	4	4	4	4	6	1.00
5	3	4	4	4	4	4	6	1.00
6	2	3	4	3	3	4	5	0.83
7	4	4	4	4	4	4	6	1.00
8	4	4	4	4	4	4	6	1.00
9	3	3	4	2	3	4	5	0.83
10	4	4	4	3	4	4	6	1.00
11	4	4	4	3	4	4	6	1.00
12	4	4	4	3	4	4	6	1.00
								^b^S-CVI/Ave = 0.97
								^c^S-CVI/UA = 0.83

^a^4-Point Rating Scale: 1 = not relevant, 2 = somewhat relevant, 3 = quite relevant, 4 = highly relevant

^b^Average scale’s content validity index

^c^Scale-level content validity index, universal agreement calculation method among experts

Table 5 summarizes the correlation between Factor-1, Factor-2 and iHOT12-Gr-total, and the selected validation instruments. The highest correlation coefficients were observed with LEFS-Gr and MHHS-Gr: 0.798 and 0.738 for Factor-1, and 0.793 and 0.725 for iHOT12-Gr-total, respectively (*p* < 0.001). The lowest correlation coefficients were between the TUG and 9S-A/D tests and Factor-2: − 0.211 and − 0.220, respectively (*p* < 0.001). These results indicate a very strong correlation between iHOT12-Gr and the PROs, while correlations with the PPMs were weak (Table 5).

**Table 5:** Construct and known-groups validity of international hip outcome tool (12 items) - Greek version (*n* = 124)

Construct validity							
		Factor-1		Factor-2		iHOT12-Gr-total^a^	
		Spearman’s correlation coefficient					
Lower Extremity Functional Scale – Greek version		0.798		0.618		0.793	
Modified Harris hip score – Greek version		0.738		0.589		0.725	
30s chair-to-stand test		0.264		0.131		0.248	
Timed Up and Go test		−0.390		− 0.211		− 0.373	
9stairs-Ascent/Descent test		− 0.388		− 0.220		− 0.383	
Known-groups validity							
Functional status^b^	N	Factor-1	*p*-value	Factor-2	*p*-value	iHOT12-Gr-total^a^	*p*-value
Poor functionality < 53	94	31.86 ± 17.85^c^	< 0.001	22.84 ± 29.45^c^	< 0.001	29.60 ± 18.83^c^	< 0.001
Good functionality > 53	30	70.46 ± 17.05^c^		48.67 ± 36.22^c^		65.01 ± 17.95^c^	

^a^The international Hip Outcome Tool (12 items) – Greek version

^b^As external criterion for examined the ability of iHOT12-Gr to distinguish subgroups of patients formed on the basis of their functional status, the cut-off point (53 points) of the Lower Extremity Functional Scale – Greek version was used. The independent samples *t*-test was used for the statistical analysis

^c^The values are expressed as Mean ± Standard deviation (SD)

The analysis of known-groups validity showed that Factor-1, Factor-2 and iHOT12-Gr-total well discriminated between subgroups of patients on the basis of their different functional status according to the cut-off of LEFS-Greek (53 points). Factor-1, Factor-2 and iHOT12-Gr-total were statistically significantly higher in participants with good functional status compared to those with poor functional status (*p* < 0.001) (Table 5).

**Fig. 5:**
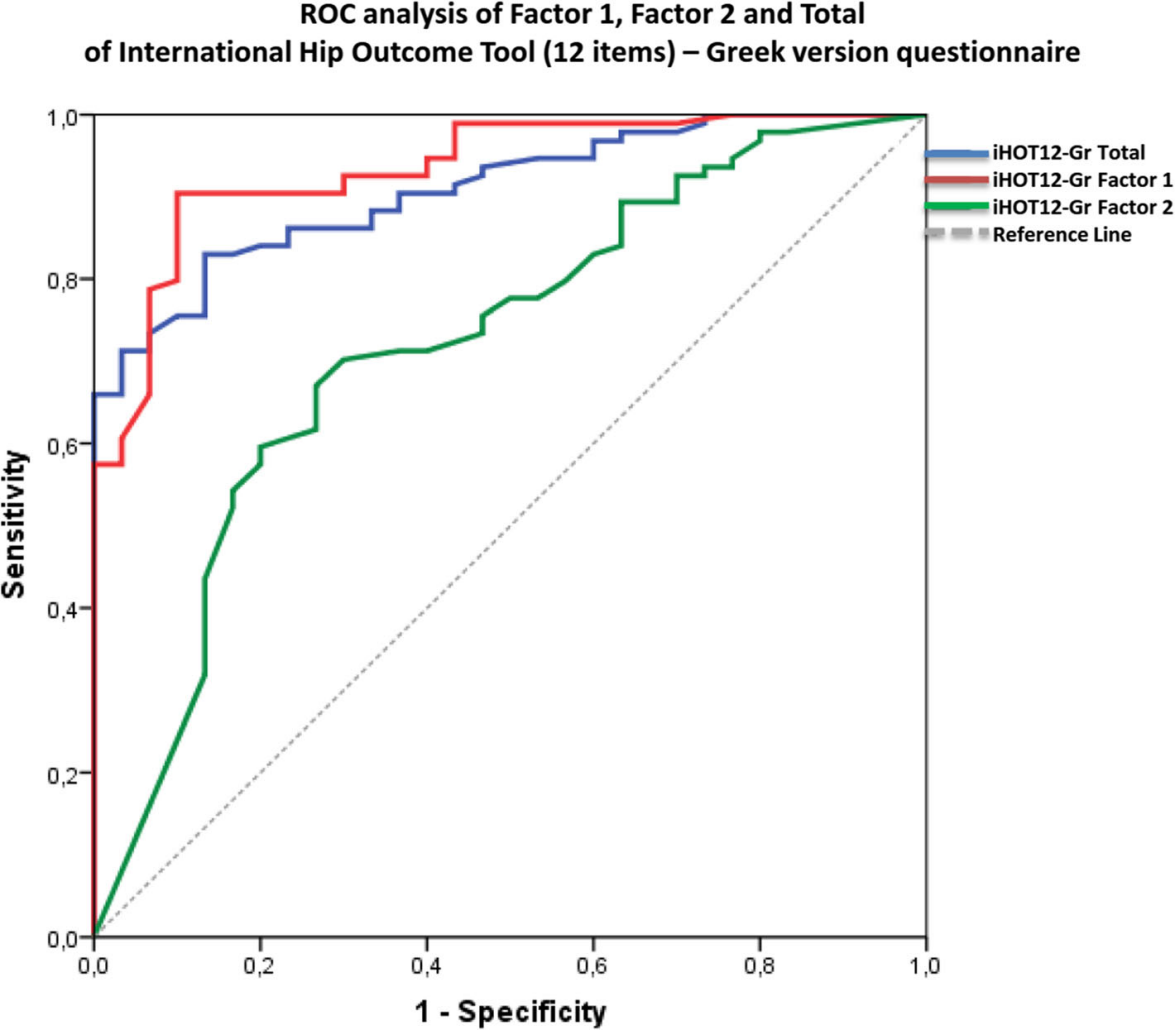
In Receiver operating curve (ROC) analysis, the area under the curve (AUC) of Factor-1 was 0.935 (95% CI 0.89–0.98 *p* < 0.001) with cut-off point 51.9, sensitivity 90% and specificity 90%. The area under the curve (AUC) of Factor-2 was 0.722(95% CI 0.61–0.80 *p* < 0.001) with cut-off point 25, sensitivity 70% and specificity 70%. The area under the curve (AUC) of iHOT12-Gr-total was 0.909 (95% CI 0.86–0.96 *p* < 0.001) with cut-off point 45.2, sensitivity 83% and specificity 87%

The ROC analysis and the cut-off points are presented in Table 6 and Fig. 5. These cut-off points show that:

- Patients who scored < 51.9 points in Factor-1 had a 90% probability of having a poor QoL, whereas patients who scored > 51.9 points had a 90% probability of having a good QoL.
- Patients with a Factor-2 score < 25 points had a 70% probability of having a poor QoL, whereas patients who scored > 25 had a 70% probability of having a good QoL.
- Patients who scored < 45.2 points in iHOT12-Gr-total had an 83% probability of having a poor QoL, whereas patients who scored > 45.2 had an 87% probability of having a good QoL.

**Table 6:** ROC analysis and cut-off point of the international hip outcome tool (12 items) – Greek version (*n* = 124)

	AUC^a^	SE^b^	*P*-value	Cut-off Point	Sensitivity	Specificity	95% CI^c^	
Factor-1	0.935	0.02	< 0.001	51.9	90%	90%	0.89	0.98
Factor-2	0.722	0.06	< 0.001	25.0	70%	70%	0.61	0.80
iHOT12-Gr-total^d^	0.909	0.03	< 0.001	45.2	83%	87%	0.86	0.96

^a^The area under the ROC curve

^b^The standard error of the area under the ROC curve

^c^confidence interval

^d^The International Hip Outcome Tool (12 items) – Greek version Total

### Responsiveness properties

There was a statistically significant increase in Factor-1, Factor-2 and iHOT12-Gr-total (all *p* < 0.001) at both the 4th and 8th postoperative week. The SRM values for all variables exceeded 0.8, so the iHOT12-Gr questionnaire showed excellent responsiveness (treatment effect validity) (Table 7).

**Table 7:** Responsiveness of the international Hip Outcome Tool (12 items) - Greek version (*n* = 25)

	Pre-operative measurement	4th postoperative week measurement	8th postoperative week measurement	*P*-value
	Mean ± SD^a^	Mean ± SD^a^	Mean ± SD^a^	
Factor-1	29.11 ± 17.70	58.94 ± 11.20^b^	78.94 ± 9.36^b,c^	< 0.001
Factor-2	22.03 ± 28.73	52.10 ± 23.27^b^	57.83 ± 22.90^b,c^	< 0.001
iHOT12-Gr-total^c^	27.34 ± 18.13	57.23 ± 11.72^b^	73.66 ± 10.87^b,c^	< 0.001
		Standardised Response Mean		
		4th postoperative week	8th postoperative week	
Factor-1		1.75	2.58	
Factor-2		1.11	1.23	
iHOT12-Gr-total^d^		1.78	2.30	

^a^Standard deviation

^b^*p* < 0.05 vs. pre-operative

^c^*p* < 0.001 vs. 4th postoperative week

^d^The international Hip Outcome Tool (12 items) – Greek version Total

## Discussion

Worldwide, this is the first study to examine the psychometric properties of iHOT12 in a sample consisting solely of patients with hip OA, and to explore and present these properties, not only for the questionnaire’s total, but also according to the 2-factor Greek model. This is also the first study to use both PROs and PPMs to examine the validity properties of iHOT12 and its responsiveness after DAA-MIS, and to determine QoL cut-off points in hip OA patients. iHOT12-Gr was found to have high/excellent reliability properties; it exhibited satisfactory validity against the LEFS-Greek and MHHS-Gr instruments, and the 30s chair-to-stand, TUG and 9S-A/D tests, and showed excellent ability to detect treatment effects.

### Factor analysis

Factor analysis of the Greek version yielded a 2-factor model: Factor-1 (items 1–9) reflects “*Symptoms and functionality*”, while Factor-2 (items 10–12) reflects “*Hip disorder-related concerns*”. The English/original version of iHOT-12 has a 4-factor model [10]. Factor analysis of the Swedish version also showed 2 factors, but with different factor loadings than ours: Factor-1 “*Function and symptoms*” (items 2–5, 8, 9) and Factor-2 “*Pain and concern/distraction*” (items 1, 6, 7, 10–12) [13]. The Dutch version showed a one-factor model [14]. The Turkish version revealed 3 factors: “*Symptom and functional limitations*” (items 1–4), “*Social, emotional and lifestyle*” (items 8–12) and “*Sports and recreational activities*” (items 6, 7, 11) [17]. These factor-model variants could be explained by cross-cultural reasons [51], or by different age-related QoL concerns and expectations, since the mean age of the studied population varied from study to study [10, 13, 14, 17]. No factor analyses have been carried out for the Portuguese, German and Japanese versions [12, 15, 16].

### Reliability

The values of Cronbach’s alpha for Factor-1 and Factor-2 were > 0.85, while the value for iHOT12-Gr-total was even higher (> 0.90), indicating that the 12 items were consistent with one another, measuring the same construct [52]. Internal consistency was not explored in the English/original version of iHOT12 [10]. The Cronbach’s alpha value for iHOT12-Gr-total is in line with the Swedish (0.90) [13], Dutch (0.96) [14], German (0.94) [15], Japanese (0.90) [16] and Turkish (0.901) [17] versions of iHOT12, confirming the questionnaire’s excellent internal consistency.

All ICC values were above the level of 0.90 (*p* < 0.001), indicating that Factor-1, Factor-2 and iHOT12-Gr-total were remarkably consistent between the two occasions. The iHOT12-Gr-total ICC values were similar to those of the English original and to those reported for other versions of iHOT12 [10, 13–17]. Hence, it could be used in clinical practice and research, since it has been reported that a PRO may be deemed adequate for use in groups (research) if the ICC is > 0.8 and for use in patients (clinical practice) if the ICC is > 0.9 [53, 54].

There was no floor or ceiling effect for the iHOT12-Gr questionnaire, consistently with other versions studied [13–15]. The absence of floor and ceiling effects is an indicative quality criterion for the questionnaire’s content validity [43].

The SEM value of iHOT-12Gr-total was found to be lower (by half) than the values from the Dutch (7.3) [14] and German (6.75) [15] versions, possibly because in those studies patients had a wider age-range (18–60 years [14] and 14–63 [15]), with a wide variety of hip pathologies, not only hip OA patients (50–85 years) as in our sample. It had been reported that a large variation in the population causes a large difference in the sample means, ultimately resulting in a larger SEM [55].

Unfortunately, our results regarding the MDC values for Factor-1, Factor-2 and iHOT-12Gr-total cannot be compared with the findings from other studies [15, 16], because we investigated MDC in terms of repeatability (the variation in repeat measurements made on the same subject under identical conditions), while the other studies explored it in terms of reproducibility (the variation in measurements made on a subject under changing conditions) [56].

### Validity

The iHOT12 questionnaire was developed to evaluate QoL in young, active patients (18–60 years old) with various hip disorders, including early hip OA [10]. In the current study, a different age range of patients (50 years and over) suffering solely from hip OA was used. Although we did not design the iHOT12, content validity evidence should be obtained to find out whether the iHOT12-Gr is suitable for our studied population [57]. This is the first iHOT12 validation study in which content validity was investigated. Item 6 (*How concerned are you about cutting/changing directions during your sport or recreational activities?*) was rated by one expert as “somewhat relevant”, while item 9 (*How much trouble do you have with sexual activity because of your hip?) *was rated similarly by another expert; probably they thought that these activities did not express the patient’s QoL. However in both items the I-CVI value was 0.83. Although the questionnaire was developed for younger populations with various hip disorders, our findings support the conclusion that individual items of iHOT12 were important and relevant to measuring QoL in elderly hip OA populations.

The construct validity results showed that Factor-1 and iHOT12-Gr-total were strongly correlated with the other PROs, while Factor-2 showed a moderate correlation. This may be explained by the fact that Factor-2 reflects “Hip disorder-related concerns” and not functionality, which LEFS-Greek and MHHS-Gr examine. Weak but significant correlations were found with the 30 s chair-to-stand, TUG and 9S-A/D tests, but this was not surprising because PPMs and PROs assess different dimensions of functionality (patient’s ability vs. patient’s perception) [58]. However, it is recommended that both are needed for a more comprehensive assessment in hip OA patients [58, 59].

Our iHOT12-Gr-total results are in line with the other iHOT12 versions. The English/original version was tested against iHOT33, showing excellent correlation [10]. The validity properties of the other versions of the iHOT12 were examined against validated cross-cultural versions of well-established questionnaires that provide generic measures of health status (i.e. EuroQol-5D, RAND 36-Item Health Survey, SF-36) or specific measures of symptoms/functionality (i.e. Copenhagen Hip and Groin Outcome Score, Hip Outcome Score, Hip disability and Osteoarthritis Outcome Score, Oxford Hip Score) [13–17]. The correlation coefficients with the generic questionnaires were moderate to strong, while those with specific questionnaires were high [13–17]. These findings indicate that iHOT12 is a valid PRO that satisfactorily captures not only the impact on QoL of the hip joint-related symptoms and functional disability, but also the related social and emotional limitations.

Known-group analysis showed that iHOT12-Gr could detect statistically significant differences in the mean scores between the study’s groups. These findings provide additional evidence for the validity of the questionnaire.

This is also the first study in which specific cut-off points for iHOT12-Gr were determined. Establishing accurate cut-off points for a PRO is of great clinical significance, given that the level of patient’s self-reported QoL influences the decision-making process and sets the goals of therapeutic intervention.

### Responsiveness

Assessment of sensitivity to change is important if a PRO is to be used in treatment evaluation studies. The large magnitude of the SRM values of Factor-1, Factor-2 and iHOT12-Gr-total at both the 4th and 8th postoperative weeks provides evidence that iHOT12-Gr is a responsive assessment with excellent ability to detect treatment effects, even soon after THA (4th week). Responsiveness has also been investigated in the English/original [10], Swedish [13], German [15] and Japanese [16] versions of iHOT12, in patients who underwent hip arthroscopy or received conservative treatment. The English/original questionnaire showed exact equivalence in responsiveness to iHOT33 [10]. In the Swedish [13] version, responsiveness was measured with an anchor based method, SRM and effect size (ES); all measures indicated large responsiveness [13]. In the German [15] and Japanese [16] versions, the criterion “*minimally important change > smallest detectable change*” [39] was used and fully satisfied. Although different statistical methods and indices were used, all studies reported that iHOT12 shows large responsiveness after conservative treatment, hip arthroscopy or DAA-MIS, as in our study.

### Strengths and limitations

This is the first study of iHOT12 in which the content validity was examined, and the first that examined the questionnaire’s properties, not only for iHOT12-Gr-total, but also for Factor-1 and Factor-2. The restrictive inclusion and exclusion criteria for participant selection from a well-defined target population are an important strength of this study. Moreover, examining the construct validity of iHOT12 against both PROs and PPMs, as well as exploring the questionnaire’s responsiveness, added statistical power to our results. However, the study also had some potential limitations. The content validity results of this study expressed the judgement of experts. Further studies should be done where content validity is evaluated by a mixed panel (experts and patients) to investigate the target group’s judgment [28]. In addition, the intraobserver reliability was not examined. Finally, since the iHOT33 questionnaire has not been officially translated into the Greek language we were unable to assess the criterion-related validity of iHOT12-Gr [6].

## Conclusion

The results shown here indicate that iHOT12-Gr has high-to-excellent reliability properties, presenting strong correlations with other PROs, significant correlations with PPMs, and showing excellent responsiveness. iHOT12-Gr could possibly be used as a joint-specific PRO in clinical practice and research to evaluate QoL in hip OA patients. Further research is needed to confirm our results and to explore the questionnaire’s properties in different groups of patients, and its responsiveness after treatments other than DAA-MIS. A broader awareness of the findings in the Greek setting would facilitate objective comparisons between studies with different national origins and could contribute to the validity of iHOT12 in future meta-analyses.

## Supplementary information

**Additional file 1.**

## Data Availability

The datasets used and/or analysed during the current study are available from the corresponding author on reasonable request.

## Abbreviations

9S-A/D: The 9-stairs-ascend/descend test
AGFI: Adjusted GFI
AMOS: Analysis of Moment Structure
AUC: Area under the curve
CFA: Confirmatory factor analysis
CFI: Comparative fit index
CI: Confidence interval
CVI: Index of content validity
d. f.: Degrees of freedom
DAA-MIS: Direct anterior approach minimally invasive surgery
EFA: Exploratory factor analysis
GFI: Goodness fit index
ICC: Intraclass correlation coefficient
I-CVI: Item-level content validity indices
iHOT12: The 12-item International Hip Outcome Tool
iHOT12-Gr: The Greek version of 12-item International Hip Outcome Tool
LEFS: The Lower Extremity Functional Scale
LEFS-Greek: The Greek version of Lower Extremity Functional Scale
MDC: Minimal detectable change
MHHS: The Modified Harris Hip Score
MHHS-Gr: The Greek version of Modified Harris Hip Score
MIC: Minimal importance change
NFI: Normed fit index
OA: Osteoarthritis
PCA: Principal component analysis
PPM: Physical-performance measure
PRO: Patient-reported outcome
QoL: Quality of life
RMSEA: Root mean square error of approximation
ROC: Receiver operating curve
S-CVI/Ave: Scale-level content validity indices average
S-CVI/UA: Scale-level content validity indices universal agreement among experts
S-CVI: Scale-level content validity indices
SD: Standard deviation
SDC: Smallest detectable change
SEM: Measurement error
SRM: Standardized response mean
THA: Total hip arthroplasty
TUG: The Timed Up & Go test
