# Supplementary figures and images for: International Hip Outcome Tool (12-items) as health-related quality-of-life measure in osteoarthritis: validation of Greek version
Source: J Patient Rep Outcomes. 2020 May 27;4:41. doi: 10.1186/s41687-020-00207-8 (PMC7253559; doi:10.1186/s41687-020-00207-8)

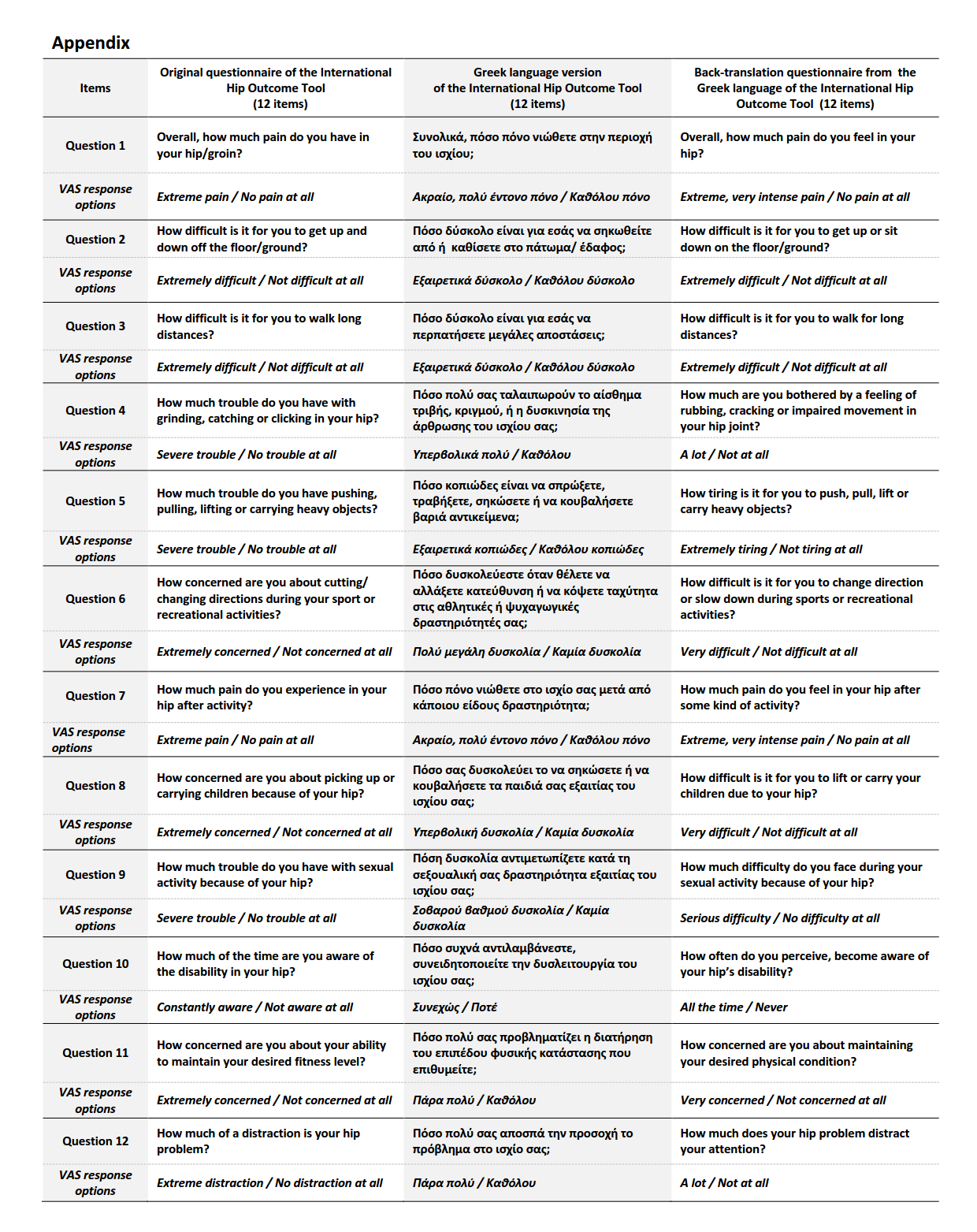

Supplement: Supplementary file 1 — Additional file 1. [file 41687_2020_207_MOESM1_ESM.png]
